# Educational

**Published:** 1892-09

**Authors:** 


### EDITORIAL.

### Educational.

In various ways is progress shown in our educational efforts. Teaching faculties are everywhere seeking and devising better methods in their work. New branches are being made tributary to our educational scheme. It is now recognized more fully, perhaps, than ever before that culture and education, both general and special, increase power, ability and influence.

The products of invention and genius are now made the property of not only the practitioner but of the student as well. The students in some dental colleges, at least, early begin a training in certain directions that practitioners have found of great value. Of these, mention may be made of students' dental societies. These have been organized in a number of colleges, and with most marked benefit to those engaging in them. Not only do they serve in fixing in the minds of the students the various subjects to which their attention is called in their regular course, but it is a training in the way of writing, speaking, and conducting association work, that not only now, but in the future will be of immense benefit. It is true that up to within a comparatively short time a very small per cent. of those who became members of dental societies took any active part in the work. Student training and association work will change this condition of things for the future; the men who are now entering our profession will in the near future make a marked change in this respect. They will be able to take their part in the conduct of such work, and, indeed, in all that pertains to it, such as writing, speaking and demonstration ; and so, we should say to students everywhere, engage in this kind of work, organize your societies in every college. It may be best sometimes for all to join in one effort, in other cases it may be better to form two or three societies, corresponding to the different classes, viz.: freshman, junior and senior. This, however, should be decided by those of each particular institution. Our advice is, therefore to the boys, organize your societies early in your college work, and continue throughout the entire course.

